# Leaf Nutrient Resorption in Lucerne Decreases with Relief of Relative Soil Nutrient Limitation under Phosphorus and Potassium Fertilization with Irrigation

**DOI:** 10.1038/s41598-020-65484-1

**Published:** 2020-06-29

**Authors:** Mei Yang, Jiaoyun Lu, Minguo Liu, Yixiao Lu, Huimin Yang

**Affiliations:** State Key Laboratory of Grassland Agro-ecosystems; Key Laboratory of Grassland Livestock Industry Innovation, Ministry of Agriculture and Rural Affairs; College of Pastoral Agriculture Science and Technology, Lanzhou University, Lanzhou, 730020 P.R. China

**Keywords:** Plant physiology, Fertilization, Abiotic, Agroecology, Grassland ecology

## Abstract

Leaf nutrient resorption is an important mechanism in adapting to adverse environments. However, few studies examine how nutrient resorption responds to phosphorus (P) and potassium (K) fertilization or to a shift in nutrient limitation due to water supply and fertilization. On the Loess Plateau of China, we treated lucerne (*Medicago sativa* L.) with P, K, or combined P and K fertilizer and three levels of water supply. The resorption efficiency of leaf P (PRE) and K (KRE) decreased with increasing water supply, whereas that of N (NRE) was unaffected. The water supply regulated the effects of P and K fertilization on resorption efficiency. With low water, P fertilization reduced NRE and significantly increased KRE. Potassium fertilization did not affect KRE and NRE, whereas PRE was significantly affected. NRE increased with increasing green leaf N:K ratio, whereas KRE and PRE decreased with increasing K:P and N:P ratios, respectively. Water supply significantly increased soil nutrient availability interactively with P or K fertilization, leading to a shift in relative nutrient limitation, which was essential in regulating nutrient resorption. Thus, lucerne growth was not limited by K but by P or by P and N, which P fertilization and water supply ameliorated.

## Introduction

Nutrient resorption from senescing leaves is a strategy for internal nutrient recycling during plant growth^1^ that reduces dependence on the soil nutrient supply^2^. Previous studies show that leaf nutrient resorption efficiency, which is the proportion of nutrients withdrawn before leaf abscission, is closely related to soil nutrient status^2,3^. Generally, leaf nitrogen (N) and phosphorus (P) resorption efficiencies (NRE and PRE, respectively) decline with increased availability of the corresponding nutrients in soil^4–6^. However, nutrient resorption efficiency is also affected by the availability of other nutrients in soils^7–9^. For example, See *et al*.^8^ found the PRE of six tree species increased with increasing soil N content in a northern hardwood forest. Wang *et al*.^9^ found that an increase in soil available P content significantly increased the K resorption efficiency (KRE) of lucerne (*Medicago sativa* L.) on the Loess Plateau of China. In addition, PRE increased with increasing soil P content in six tree species in a northern hardwood forest^8^ but only changed marginally for the palm (*Oenocarpus mapora*) in a lowland tropical forest^10^. The inconsistent responses of nutrient resorption to soil nutritional status have thus encouraged efforts to explain why resorption changes in different ways with the availability of other nutrients.

Nutrient resorption efficiency is regulated not only by the absolute content of individual nutrients but also by the balance among various nutrients in soil^11,12^. Both fertilization and water supply can affect leaf nutrient resorption via changing the availability of individual nutrients, but the responses of resorption are inconsistent. Generally, P fertilization leads to a decrease in PRE because of increased soil P availability^5,6^. However, in other studies, PRE increases or changes little with P fertilization^8,10^. Water supply changes soil nutritional status, which also leads to different resorption responses^2,12–14^. Generally, nutrient mineralization increases with an increase in soil water because of increased activities of P-transforming enzymes and nitrogenase in N_2_-fixing nodules^15,16^. Such inconsistent results suggest that some other mechanisms, rather than only change in individual nutrients, are involved in regulating nutrient resorption. Güsewell^11^ analyzed a global data set of woody plants and found that NRE and PRE were, on average, higher in P-limited than in N-limited sites. Tang *et al*.^17^ further found that woody plants under P limitation had higher PRE than those under N limitation, whereas under N limitation, the NRE was higher than that under P limitation. In a meta-analysis across multiple scales, Reed *et al*.^12^ concluded that the N:P resorption ratio could be an indicator of soil nutrient limitation. In these previous studies, leaf stoichiometric ratios of nutrients provide some clues on the regulation of nutrient resorption under relative soil nutrient limitation^6,18^. However, a clear understanding of how resorption changes with a shift in nutrient limitation remains elusive.

Fertilization increases the availability of applied nutrients but can change relative nutrient limitations by affecting the availability of other nutrients in soils^7,18^. For example, in N-limited ecosystems, P limitation gradually increases after N fertilization or atmospheric deposition because plant growth is promoted, which increases P demand^19^. In addition, soil acidification caused by N enrichment can also restrict mycorrhizal activity and thus P mineralization^20,21^, thereby reducing soil P availability. However, P availability can also increase in soils enriched with N, because P-transforming enzyme activity is stimulated^22^. Therefore, N fertilization can affect P limitation in soils in various ways. Similarly, P fertilization can increase N-limitation in plants at P-limited sites^23^ or promote N availability by stimulating biological N_2_ fixation (BNF) in legume species^24^. Furthermore, when soil N and P are enriched, K limitation occurs in bogs^25^. Thus, further investigation is needed to reveal how fertilization and water supply affect relative nutrient limitations and how any changes affect leaf resorption.

The Loess Plateau of China is well known for serious soil erosion^26^, which leads to soil infertility. In addition, the seasonal imbalance in precipitation in this region leads to great variation in soil water availability^27^, resulting in either reduced nutrient availability because of drought or nutrient loss with occasional storm-induced soil erosion. Lucerne is widely sown and performs well on the plateau because of its strong BNF and broad tolerance to adverse environments^28^. However, the productivity and sustainability of lucerne grasslands are still affected by limitations in soil nutrients and water availability^14^. Leaf nutrient resorption plays an important role in lucerne adaptation to this environment^9,14^. An increase in the water supply can increase leaf PRE in lucerne but has little effect on NRE^14^, which helps the growth and production of this forage. In practice, P and K fertilizers are typically applied to sustain lucerne production on the plateau; whereas the use of N fertilizer is limited because of BNF. Phosphorus fertilization increases NRE and KRE of lucerne on the plateau but affects PRE in various ways^9,28^, whereas N fertilization has little effect on NRE and KRE but tends to increase PRE^28^. However, the effects of water supply on leaf nutrient resorption of lucerne under P and K fertilization remain uncertain. Compared with other species, legumes may generally have a greater demand for other nutrients than N because of strong BNF, which possibly changes the balance between N and other nutrients and thus causes a shift in relative nutrient limitation. Therefore, strong resorption of nutrients other than N may help legumes to better grow and survive.

In this study, we tested the hypothesis that leaf nutrient resorption would decrease under an increase in water supply and fertilizer application, both of which can relieve relative nutrient limitations in soil. The objectives of this study were to determine the following: (i) how leaf NRE, PRE, and KRE of lucerne change with P and K fertilization at different levels of water supply; (ii) how relative nutrient limitation is affected by P and K fertilization and the water supply; and (iii) how leaf nutrient resorption responds to shifts in relative nutrient limitations.

## Results

### Changes in leaf nutrient resorption efficiencies under phosphorus and potassium fertilization and three levels of water supply

**Figure 1:**
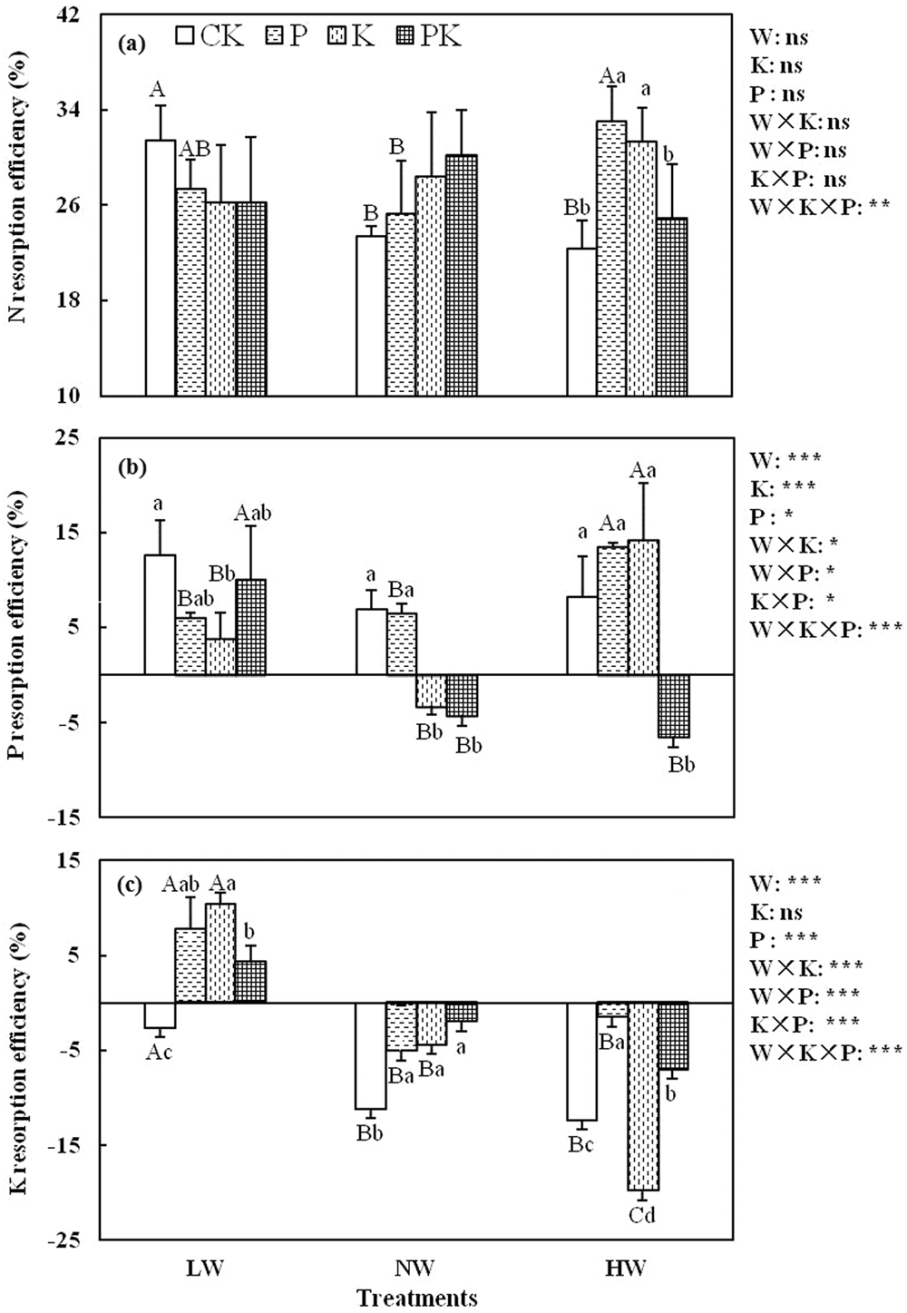
Effects of P and K fertilization on (**a**) N, (**b**) P, and (**c**) K resorption efficiencies of lucerne under three water supply levels. CK, no P or K fertilization; LW, low water supply (300 mm year^−1^); NW, normal water supply (450 mm year^−1^); HW, high water supply (600 mm year^−1^). Water was supplied in flood irrigation. Different capital letters denote significant differences among water supply levels under the same fertilization treatment (*p* < 0.05). Different lowercase letters denote significant differences among fertilization treatments under the same water supply level (*p* < 0.05). There are no significant differences among water supply levels and fertilization treatments, the letters are not shown (*p* > 0.05). The data are presented as the mean ± SD (n = 36). From three-way ANOVA, effects of water supply (W), P fertilization (P), K fertilization (K) and their interactive effects are shown on the right side. *, *p* < 0.05; **, *p* < 0.01; ***, *p* < 0.001; ns, not significant.

Phosphorus fertilization and water supply significantly affected PRE and KRE (*p* < 0.05; Fig. 1b,c), and K fertilization significantly affected PRE (*p* < 0.001), but NRE was not affected by P and K fertilization or water supply (*p* > 0.05; Fig. 1a). The effects of P and K fertilization were regulated by water supply (Fig. 1). Compared with the CK (without fertilization), P fertilization did not significantly affect PRE at any water supply level. By contrast, P fertilization increased NRE only under HW (high water supply), whereas KRE increased under all levels of water supply (Fig. 1). Potassium fertilization significantly increased NRE only under HW (*p* < 0.05), whereas PRE decreased under LW and NW (low and normal water supply levels, respectively) but tended to increase under HW. The effects of K fertilization on KRE were opposite to those on PRE (Fig. 1). The P and K co-fertilization significantly decreased PRE under NW and HW but increased KRE under the different levels of water supply (*p* < 0.05; Fig. 1b,c).

### Changes in soil nutrient contents and relative nutrient limitation under phosphorus and potassium fertilization and three levels of water supply

Phosphorus fertilization significantly affected the contents of soil nitrate N and total and available K (*p* < 0.05), and K fertilization significantly increased total N content (*p* < 0.05; Table 1). Water supply significantly increased the contents of nitrate N, ammonium N, total P, total K (*p* < 0.01), and available P (*p* < 0.05; Table 1). The effect of fertilization was regulated by water supply in some cases (Table 1). Compared with the CK, The nitrate N content increased significantly under P fertilization and LW and HW (*p* < 0.05; Table 1). Phosphorus and K co-fertilization significantly decreased ammonium N and available K under NW (*p* < 0.05; Table 1).

**Table 1:** Total and available soil nutrients contents under P and K fertilization under three water supply levels and the effects of water supply (W), P fertilization (P), K fertilization (K) and their interaction on soil total and available nutrients contents based on three-way ANOVA.

Water availability	Fertilization	Total N (kg m^−2^)	Nitrate N (g m^−2^)	Ammonium N (g m^−2^)	Total P (kg m^−2^)	Available P (g m^−2^)	Total K (kg m^−2^)	Available K (kg m^−2^)
LW	CK	0.59^a^	2.63^Cb^	0.73	0.48^B^	20.1^b^	8.17^Cb^	125
	P	0.59^Ba^	4.19^Ba^	0.77^C^	0.59	19.0^b^	7.93^Bb^	133
	K	0.67^ab^	4.25^Ba^	0.63^C^	0.49^B^	16.9^Bb^	7.94^Cb^	150
	P + K	0.71^b^	4.69^Ba^	0.69^B^	0.68	27.8^Aa^	8.82^a^	165^A^
NW	CK	0.70	4.79^B^	1.10^b^	0.45^B^	15.5^ab^	8.67^Ba^	151^b^
	P	0.68^AB^	3.53^B^	1.50^Aa^	0.60	21.9^a^	8.71^Aa^	154^b^
	K	0.72	5.37^B^	1.71^Aa^	0.63^A^	20.4^ABa^	8.71^Ba^	202^a^
	P + K	0.63	4.15^B^	0.81^Bc^	0.57	13.5^Cb^	8.21^b^	116^Bc^
HW	CK	0.63^b^	10.32^Ab^	0.98	0.60^A^	18.7^b^	9.46^Aa^	166
	P	0.72^Aab^	18.18^Aa^	1.18^B^	0.68	18.9^b^	9.08^Aa^	156
	K	0.81^a^	11.10^Aab^	1.14^B^	0.76^A^	26.2^Aa^	9.27^Aa^	195
	P + K	0.71^ab^	16.20^Aab^	0.97^A^	0.66	20.5^Bab^	8.37^b^	148^AB^
P		NS	*	NS	NS	NS	*	*
K		*	NS	NS	NS	NS	NS	NS
W		NS	***	***	**	*	***	NS
W × P		NS	**	*	NS	*	***	*
W × K		NS	NS	NS	NS	NS	***	NS
P × K		NS	NS	***	NS	NS	NS	*
W × P × K		NS	NS	***	NS	***	***	NS

Soil nutrient content represented the entire 0–60 cm profile. LW, low water supply (300 mm year^−1^); NW, normal water supply (450 mm year^−1^); HW, high water supply (600 mm year^−1^); CK, no P or K fertilization. Different capital letters denote significant differences among water supply levels under the same fertilization treatment (*p* < 0.05). Different lowercase letters denote significant differences among fertilization treatments at the same water supply level (*p* < 0.05). There are no significant differences among water supply levels and fertilization treatments, the letters are not shown (*p* > 0.05). The data are presented as the mean ± SD (n = 36). ***, *p* < 0.001; **, *p* < 0.01; *, *p* < 0.05 and NS, no significance.

**Figure 2:**
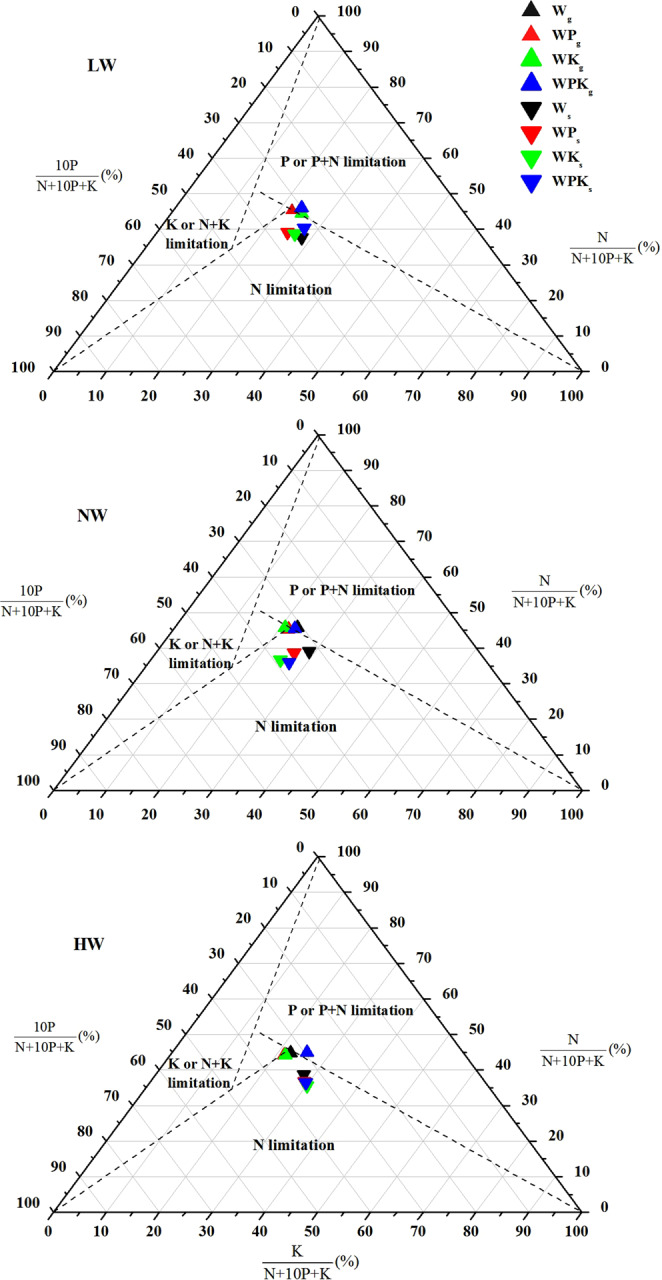
Ternary diagrams showing N, P, and K stoichiometric relationships in green (delta up and subscript *g*) and senesced (delta down and subscript *s*) leaves of lucerne in response to P and K fertilization under three water supply levels: LW (top, low water, 300 mm year^−1^), NW (middle, normal water, 450 mm year^−1^), HW (bottom, high water, 600 mm year^−1^). Water was supplied in flood irrigation. Dashed lines indicate the critical ratios of N:P (14.5), N:K (2.1), and K:P (3.4), dividing the plots into four parts, of which three indicate N limitation (N:P < 14.5 and N:K < 2.1), P limitation or P + N co-limitation (N:P > 14.5 and K:P > 3.4), and K limitation or K + N co-limitation (N:K > 2.1 and K:P < 3.4). In the central triangle section, the stoichiometric ratio cannot be used to determine the type of nutrient limitation or there is no NPK limitation^55^. For visual reasons, the P concentration was multiplied by 10. LW + P fertilization, LWP; LW + K fertilization, LWK; LW + P and K co-fertilization, LWPK; NW + P fertilization, NWP; NW + K fertilization, NWK; NW + P and K co-fertilization, NWPK; HW + P fertilization, HWP; HW + K fertilization, HWK; HW + P and K co-fertilization, HWPK.

In all treatments, neither green nor senesced leaves were within the K-limited section of the ternary diagrams and were not primarily K-limited even after P and K fertilization at the three water supply levels (Fig. 2). The effect of P fertilization on N:P:K stoichiometry was more substantial in green leaves than in senesced leaves, whereas the effects of K fertilization and water supply were more substantial in senesced leaves (Table 2). In green leaves, there were significant increases in the relative P concentrations under K fertilization, but no change was observed in the relative N concentration with the increase in water supply (*p* < 0.05). The P and K co-fertilization decreased the relative P concentration and increased the relative K concentration in green leaves under HW (Table 2; Fig. 2). In senesced leaves, there were increases in the relative P and K concentrations with increasing water supply but a decrease in the relative N concentration. In addition, there were significant increases in the relative P concentration (*p* < 0.05) and decreases in the relative N and K concentrations under fertilization in senesced leaves under NW (Table 2; Fig. 2). Phosphorus and K co-fertilization generally decreased the relative P concentration and increased the relative N concentration in senesced leaves under LW. With P, K, and P and K co-fertilization, there was no significant change in relative P concentration in senesced leaves under HW (Table 2; Fig. 2). Therefore, fertilization and water supply regulated the relative nutrient limitations in soil, and lucerne growth was primarily limited by soil P and N.

**Table 2:** Specific N, P, and K stoichiometric ratios in green and senesced leaves of lucerne and effects of water supply (W), P fertilization (P), K fertilization (K) and their interaction on the ratios based on three-way ANOVA.

Water availability	Fertilization	Green leaf			Senesced leaf		
		$\frac{{\bf{N}}}{{\bf{N}}{\boldsymbol{+}}{\bf{10}}{\bf{P}}{\boldsymbol{+}}{\bf{K}}}{\boldsymbol{(}}{\boldsymbol{ \% }}{\boldsymbol{)}}$	$\frac{{\bf{10}}{\bf{P}}}{{\bf{N}}{\boldsymbol{+}}{\bf{10}}{\bf{P}}{\boldsymbol{+}}{\bf{K}}}{\boldsymbol{(}}{\boldsymbol{ \% }}{\boldsymbol{)}}$	$\frac{{\bf{K}}}{{\bf{N}}{\boldsymbol{+}}{\bf{10}}{\bf{P}}{\boldsymbol{+}}{\bf{K}}}{\boldsymbol{(}}{\boldsymbol{ \% }}{\boldsymbol{)}}$	$\frac{{\bf{N}}}{{\bf{N}}{\boldsymbol{+}}{\bf{10}}{\bf{P}}{\boldsymbol{+}}{\bf{K}}}{\boldsymbol{(}}{\boldsymbol{ \% }}{\boldsymbol{)}}$	$\frac{{\bf{10}}{\bf{P}}}{{\bf{N}}{\boldsymbol{+}}{\bf{10}}{\bf{P}}{\boldsymbol{+}}{\bf{K}}}{\boldsymbol{(}}{\boldsymbol{ \% }}{\boldsymbol{)}}$	$\frac{{\bf{K}}}{{\bf{N}}{\boldsymbol{+}}{\bf{10}}{\bf{P}}{\boldsymbol{+}}{\bf{K}}}{\boldsymbol{(}}{\boldsymbol{ \% }}{\boldsymbol{)}}$
LW	CK	45.23	32.23a	22.54b	37.67b	34.22Aab	28.11a
	P	45.21	32.29a	22.50ABb	39.12Aab	36.16a	24.72Bc
	K	44.49	30.75Bb	24.76Aa	38.75Aab	35.02Bab	26.23Bbc
	P+K	45.93	30.12b	23.95Bab	40.34Aa	32.34Bb	27.32Bab
NW	CK	45.73	30.97	23.30a	39.04a	32.10Bc	28.86a
	P	45.27	32.89	21.84Bb	38.62ABa	35.19b	26.19Bb
	K	45.78	33.30A	20.92Bc	36.76ABb	38.69Aa	24.54Cc
	P+K	45.51	31.55	22.94Ba	36.05Bb	37.36Aab	26.59Bb
HW	CK	44.83	32.67a	22.50ab	38.67a	33.26AB	28.07b
	P	44.59	32.05a	23.35Ab	36.74Bab	34.10	29.16Aab
	K	44.30	34.10Aa	21.60Bc	35.58Bb	34.18B	30.24Aa
	P+K	44.96	29.49b	25.54Aa	36.45Bab	33.99AB	29.56Aab
P		NS	*	**	NS	NS	NS
K		NS	NS	*	*	*	NS
W		NS	NS	**	***	**	***
W × P		NS	**	***	*	NS	NS
W × K		NS	*	**	**	***	***
P × K		NS	***	***	NS	**	***
W × P × K		NS	NS	**	NS	NS	***

LW, low water supply (300 mm year^−1^); NW, normal water supply (450 mm year^−1^); HW, high water supply (600 mm year^−1^); CK, no P or K fertilization. Different capital letters denote significant differences among water supply levels under the same fertilization treatment (*p* < 0.05). Different lowercase letters denote significant differences among fertilization treatments at the same water supply level (*p* < 0.05). There are no significant differences among water supply levels and fertilization treatments, the letters are not shown (*p* > 0.05). The data are presented as the mean ± SD (n = 36). ***, *p* < 0.001; **, *p* < 0.01; *, *p* < 0.05 and NS, no significance. For visual reasons, the P concentration was multiplied by 10.

### Relationship of nutrient resorption with soil nutrient availability

**Figure 3:**
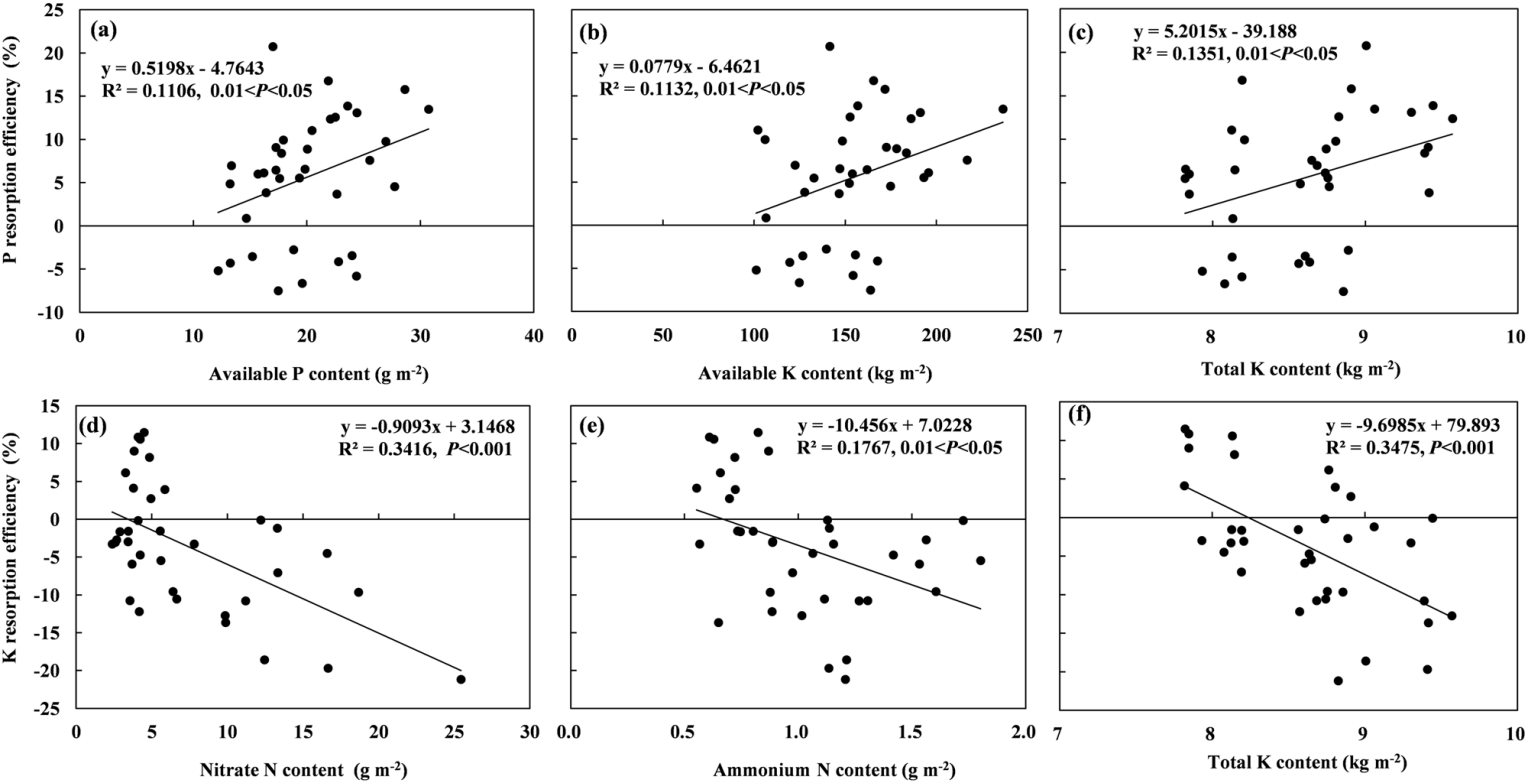
Linear relations (*y* = a*x* + b) between (**a–c**) P and (**d–f**) K resorption efficiencies and soil nutrient contents. All data are used for identifying the correlations using Spearman’s rank correlation analysis. Only significant cases are shown in the figures (*p* < 0.05; n = 36).

The PRE was significantly positively correlated with the contents of available P and total and available K in soils (Fig. 3a–c). By contrast, KRE was significantly negatively correlated with the contents of nitrate N, ammonium N, and total K (Fig. 3d–f). No significant regressions occurred between NRE and soil nutritional status.

**Figure 4:**
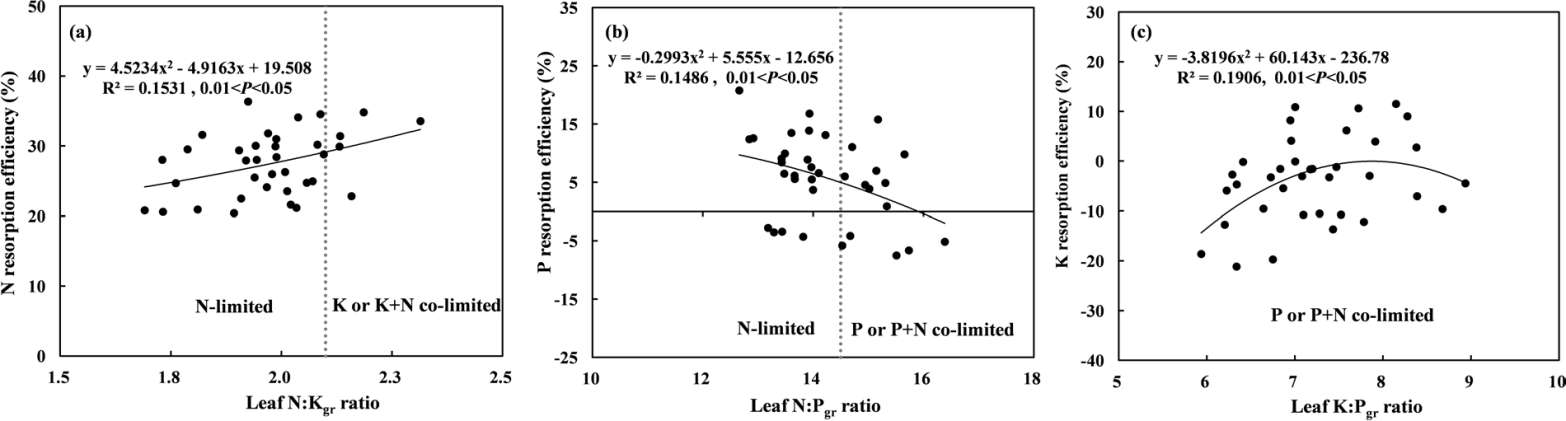
Quadratic relations (*y* = a*x*^2^ + b*x* + c) between the (**a**) N, (**b**) P, and (**c**) K nutrient resorption efficiencies of lucerne and green leaf stoichiometry: (**a**) N:K, (**b**) N:P, and (**c**) K:P. All data are used for identifying the correlations using Spearman’s rank correlation analysis. Only significant cases are shown in the figures (*P* < 0.05; n = 36). *gr*, green leaf. Dashed lines indicate the critical ratios of (**a**) N:K (2.1) and (**b**) N:P (14.5), showing N limitation (N:K < 2.1 and N:P < 14.5), P limitation or P + N co-limitation (N:P > 14.5 and K:P > 3.4), and K limitation or K + N co-limitation (N:K > 2.1 and K:P < 3.4)^55^. In this study, the values of K:P were much higher than 3.4, for the sake of graphics, the dashed line indicating the critical K:P ratio were not added.

There were a few significant correlations of resorption efficiencies with stoichiometric ratios of green leaves that varied with the shifts in relative nutrient limitations of soils (Fig. 4). When the soil was N-limited, NRE increased with the increase in the proportional amount of N compared with that of K in green leaves (N:K_gr_). In general, NRE in K limited or K + N co-limited soils was higher than that in N-limited soils (Fig. 4a). When the soil appeared to be N- or P-limited or P + N-co-limited, PRE decreased with the increase in the proportional amount of N compared with that of P in green leaves (N:P_gr_). The PRE in N-limited soils was higher than that in P-limited or P + N-co-limited soils (Fig. 4b). When the soil was P-limited or P + N-co-limited, KRE mostly increased with the increase in the proportional amount of K compared with that of P in green leaves (K:P_gr_). When the proportional amount of K compared with that of P in green leaves (K:P_gr_) was < 7.87, KRE increased with K:P_gr_, whereas KRE decreased with K:P_gr_ when K:P_gr_ > 7.87 (Fig. 4c).

## Discussion

Nutrient resorption efficiency is different at different intensities of drought stress or levels of soil water^12^. Generally, nutrient resorption efficiency increases with drought stress^29^ or with a reduction in precipitation or water supply^2^, whereas the addition of water can lead to a decrease in NRE but an increase in PRE^13^. In this study, PRE and KRE both decreased with increasing water supply, whereas NRE was largely unaffected (Fig. 1). An increase in soil water should lead to increased availability of P and K in soils or to reduced relative limitations of P and K compared with N in soils, resulting in the reduction in P and K resorption. By contrast, BNF in lucerne increases total N and availability of soil mineral N with or without a change in water supply. Therefore, NRE was relatively stable.

Phosphorus fertilization leads to various responses in nutrient resorption efficiency in different environments^5,6,8,10,23^. Generally, P fertilization leads to a decrease in PRE because of increased soil P availability^5,6^. In this study, although P fertilization had few effects on PRE, the water supply strongly affected PRE (Fig. 1b). Under a low water supply, P fertilization-induced increases in soil P availability can satisfy plant growth needs, although the increase in growth may be limited because of the restricted solubility and mobility of inorganic P in arid soils^30^. Thus, the need for P resorption is reduced. By contrast, an adequate supply of water greatly promotes plant growth, resulting in an increased requirement for P, especially in green, active leaves. However, the poor solubility and mobility of P in soils reduce the increase in soil P availability induced by P fertilization^31^, which ultimately led to diluted concentrations of P in leaves with increasing leaf area (Fig. S1a) and thus more P removed from the senesced leaves. Therefore, P fertilization reduced PRE under LW, whereas the opposite effect was observed under HW. Additionally, the effect of P fertilization on NRE was also regulated by water supply. In this study, P fertilization reduced NRE under LW (Fig. 1a). This result might be explained by an increase in the N source due to increased BNF, which increases because the P input in fertilizer increases the activity of N_2_-fixing nodules^32^. By contrast, a high water supply level not only promotes BNF but also increases mineral N, and an increase in mineral N tends to retard BNF; thus, total N use is restricted, leading to a P fertilization-induced increase in NRE^16^. Phosphorus fertilization significantly increased KRE, which is consistent with the finding of Wang *et al*.^9^ This result might be attributed to an increase in the requirement for K when P fertilization promoted plant growth (Fig. S1b).

The effects of K fertilization are different from those of P fertilization. Potassium is highly mobile in the soil–plant system and is generally at high concentration in the experimental site and therefore can meet the requirement for plant growth with or without K fertilization. Wright and Westoby^33^ found that the proportion of resorbed vs. soil-derived nutrients deployed in new leaves of Australian sclerophyll species was set by the relative cost of obtaining nutrients from the two sources. Therefore, it may cost less energy to absorb K from soils than to reabsorb K from senesced leaves when there is sufficient K. This relation could explain why, in this study, K fertilization had little effect on KRE (Fig. 1). However, K fertilization significantly affected PRE but had little effect on NRE, which was also affected by water supply (Fig. 1). Yan *et al*.^34^ reported that leaf K concentration was closely related to water availability. In this study, we found leaf K concentration increased significantly with the increase in water supply (Fig. S1a,d). Thus, the increase in water availability might have promoted K uptake.

Water is beneficial to nutrient mineralization^16^, because an increase in soil water may increase the activities of related enzymes, such as P-transforming enzymes and nitrogenase in N_2_-fixing nodules. In this study, the contents of total and available P, available N, and total K increased significantly with the increase in water supply (Table 1), suggesting that the increase in water supply increased soil nutrient availability. Fertilization usually increases the availability of soil mineral nutrients, especially of the corresponding nutrients^5,6^. However, fertilization does not always increase the availability of soil nutrients, and in this study, we did not detect significant changes in most nutrients (Table 1). The immobility of P in soils may lead to less variation in available P content during its release^30,31^. By contrast, soil K is highly mobile and therefore moves downward quickly, resulting in less accumulation in the shallower soils (Table 1). Phosphorus and K fertilization did not change the contents of N in lucerne soil because of a lack of regulation of BNF. In addition, the water supply, to some extent, increased the effects of P and K fertilization on available nutrients because it promoted solubility and mineralization.

Alteration of the stoichiometry of plant tissues can imply a change in soil nutrient limitation^6,11^. However, there is no consensus on the tissue in which stoichiometry best indicates changes in relative soil nutrient limitation^35,36^. In this study, the effect of P fertilization on N:P:K stoichiometry was more important in green leaves than in senesced leaves, whereas the effects of K fertilization and water supply were more important in senesced leaves (Table 2). These results showed that fertilization and water supply variously affected the relative limitations of soil nutrients and that green and senesced leaves showed contrasting responses to those limitations^37^. The relative P concentration increased with K fertilization in green leaves and with the increase in water supply in senesced leaves (Table 2; Fig. 2), and thus, leaf nutrient stoichiometry indicated amelioration of P limitation. In addition, the stoichiometry indicated that changes in relative concentrations in senesced leaves were most regulated by water supply. In senesced leaves, P, K, and P and K co-fertilization generally increased the relative N concentration and decreased the relative P concentration under LW, but decreased the relative N concentration and increased the relative P concentration under NW, and did not substantially change the relative P concentration under HW (Table 2; Fig. 2). Combined with the results from senesced leaves; plant growth was primarily limited by N (Fig. 2). Therefore, when soil water is at a deficit, fertilization may not relieve the N-limitation but increase the P limitation. Whether on the basis of the nutrient stoichiometry of green or senesced leaves, the growth of lucerne was not limited by K but by P or by P and N (Fig. 2). No other evidence has indicated relative K limitation in the study area. By contrast, Lu *et al*.^14^ found the growth of lucerne was affected by relative P limitation in loess soils of China, in addition to N.

Generally, nutrient resorption efficiency is higher when soil nutrient levels are lower^4,5,38^ and decreases with increased availability of corresponding nutrients in soils^4–6^. However, inconsistent results are reported from diverse studies^9,39^. In this study, NRE had no relationship with soil nutritional status. However, NRE increased with an increase in the proportional amount of N compared with that of K in green leaves (N:K_gr_) under N- and K-limited or K + N-co-limited soils, and the NRE in K-limited or K + N-co-limited soils was higher than that in N-limited soils (Fig. 4a). These results may be explained because K^+^ is absorbed as a companion ion of NO_3_^−^ during migration from soil to plant root and as a univalent cation, can balance anions in the xylem and endodermis during transport^40,41^.

In previous studies, PRE either increased^29^ or was little affected by decreasing P availability in soils^42,43^. In this study, PRE was positively correlated with soil available P content (Fig. 3a). Phosphorus uptake can be limited despite high soil available P^35^, because N and P co-addition can affect the uptake of plant nutrients and water by reducing the abundance of arbuscular mycorrhizal fungi^44^. As a result, P resorption increases even with high P availability. Zhou *et al*.^45^ found that PRE in green leaves decreased with an increase in the proportional amount of N compared with that of P (N:P_gr_) or with a decrease in the proportional amount of K compared with that of P (K:P_gr_). In this study, PRE decreased with an increase in the proportional amount of N compared with that of P (N:P_gr_) in N- and P-limited or P + N-co-limited soils, and the PRE in N-limited soils was higher than that in P-limited or P + N-co-limited soils (Fig. 4b). Killingbeck^46^ proposed that nutrient concentration thresholds occur in senesced leaves that determine complete or incomplete resorption of N and P. Han *et al*.^36^ found that woody plants with N limitation tended to have complete N resorption but incomplete P resorption and with N + P co-limitation tended to have incomplete or intermediate resorption of both N and P. In this study, the nutrient concentrations in senesced leaves are consistent with these results, except for the incomplete N resorption with N limitation (Fig. S2). Additionally, P limitation or P + N co-limitation can increase P uptake from roots because of the higher energy consumption during resorption than during root uptake^47^, and thus, in this study, PRE continuously declined from N limitation to P limitation or P + N co-limitation (increasing N:P_gr_). We also found that PRE was positively correlated with available and total K contents (Fig. 3b,c). In plants, K plays a key role in maintaining cell turgidity and integrity during growth and survival, and a high level of K helps to sustain plant functions during rapid growth^41^, which requires more P, leading to an increase in PRE.

In this study, KRE was significantly negatively correlated with the contents of nitrate N, ammonium N, and total K in soils (Fig. 3d–f). In addition, when the proportional amount of K compared with that of P (K:P_gr_) in green leaves was < 7.87, KRE increased with increasing K:P_gr_, whereas when K:P_gr_ was > 7.87, KRE decreased with increasing K:P_gr_ (Fig. 4c). Plants absorb high-affinity K^+^ via K^+^ transporters when the external K^+^ is lower than 0.2 mM, whereas when the external K^+^ is higher than 0.3 mM, plants absorb low-affinity K^+^ through K^+^ channels^48^. In this study, the changes in KRE with increasing K:P_gr_ might also be regulated by these dual mechanisms. Additionally, K^+^ is synergistically transported with PO4^3-^^40,41^, resulting in increased KRE under relative P limitation.

In summary, lucerne growth was not limited by K but by P or by P and N on the Loess Plateau of eastern Gansu Province, China, which can be ameliorated by P fertilization and water supply (precipitation or irrigation). The water supply interactively affects the availability of soil nutrients with fertilization, leading to a shift in the relative nutrient limitation of soils. Phosphorus and K fertilization showed contrasting effects on leaf N, P, and K resorption efficiencies, which were regulated by water supply. Relative nutrient limitation, in addition to the absolute content of individual nutrients in soils, is essential in regulating leaf nutrient resorption in lucerne.

## Materials and methods

### Study site description

The study was conducted at the Qingyang Loess Plateau Pastoral Agriculture Station (35°40′N, 107°51′E; 1,298 m above sea level), which is an experimental and educational station of Lanzhou University in Qingyang, Gansu Province, China. The climate is typical continental, and the mean annual temperature and precipitation are 9.2 **°**C and 543 mm, respectively, with 70% of total precipitation concentrated in July, August, and September. The soil (locally, Heilu soil) is described as an Entisol in the classification of the Food and Agriculture Organization of the United Nations, a sandy loam contains 70% silt and 23% clay, and represents the major cropping soil found in the region^8^. The area of the station is 14.67 ha. Winter wheat (*Triticum aestivum* L.), soybean (*Glycine max* Merr.), forage maize (*Zea mays* L.), and lucerne, among others, are cultivated on this station and in the surrounding around areas.

### Experimental design

The experiment was conducted under rain shelters that kept rainfall from the test plots. The shelters had a steel frame covered by transparent plastic film that allowed photosynthetically active radiation to pass for lucerne growth. The experiment was a randomized complete block factorial design with water supply and P or K fertilization as the factors. Three levels of water supply based on the local average rainfall over the years were set as follow: low (LW, 300 mm year^−1^), normal (NW, 450 mm year^−1^), and high (HW, 600 mm year^−1^), with water supplied as irrigation. Four fertilization treatments were set as follow: without P and K fertilization (CK), P fertilization (P), K fertilization (K), and P and K co-fertilization (P + K). Each treatment had three replicates, and thus, there were 36 plots (4 m × 2 m) under the shelters. On 12 April 2016, a landrace of lucerne (*M. sativa* L. ‘Longdong’) was sown in rows at the rate of 22.5 kg ha^−1^. Nitrogen fertilizer was applied as granulated urea at the rate of 7.5 g N m^−2^ to all plots, which was according to the usual amount of fertilizer applied locally. Phosphorus fertilizer was applied to P plots as granulated calcium superphosphate (containing 16% P_2_O_5_) at the rate of 10 g P m^−2^. Potassium fertilizer was applied to K plots as granulated potassium sulfate (containing 51% K_2_O) at the rate of 4.65 g K m^−2^. The plots were fertilized at sowing. Flood irrigation was applied once about every 20 days and ten times per year in all water treatments, with irrigation usually performed in the morning or late afternoon to reduce water loss through evapotranspiration.

### Sampling

On 18 September 2016, soil samples (0–10, 10–20, 20–30, and 30–60-cm depths) were randomly collected with two soil cores (5-cm diameter) in each plot. Roots and all organic debris were removed by hand from the samples, which were then air-dried and passed through a 2-mm sieve to measure nitrate N, ammonium N, available K, and available P or a 0.25-mm sieve to measure total N, total K, and total P.

Lucerne is perennial forage and is generally cut three times per year in the area. In each cut, lucerne is cut at the early flowering stage, much earlier than the end of the growing season when most of the leaves would turn yellow or brown. In this case, a leaf could be marked at the peak growing time of lucerne but the senesced marked leaf would not be available at the sampling date^49^. Therefore, on 14 August 2016, green and senesced leaves of lucerne were simultaneously sampled when the stands were at the early flowering stage (second cut)^50^ in this study. At least 15 shoots were chosen in each plot, and at least 10 green leaves that were fully expanded and 10 senesced leaves that were withered and brown but still attached to the stem were taken from each shoot. Leaf samples were then oven-dried at 65 °C for at least 48 h until constant weight. Dried samples were ground uniformly and passed through a 1.0-mm sieve to measure N, P, and K concentrations.

### Measurements

Soil bulk density was determined in each plot by randomly taking two soil cores at each 10-cm depth from 0 to 60 cm. The cores were taken by vertically pounding stainless steel cylinders (height, 52 mm; diameter, 70 mm; 200 cm^3^ inner volume) with a cutting edge into the middle of each soil layer with a hammer. The cores were stored and transported in aluminum cans to determine gravimetric water content and then dried and weighed to compute soil bulk density.

Total N concentrations of plant and soil were determined using a semimicro-Kjeldahl method with a Kjeltech 8400 Analyzer Unit (FOSS, Sweden). Soil nitrate N content was measured using an ultraviolet spectrophotometer method (UV-2102 PCS, Shanghai, China). Soil ammonium N was extracted in 2 mol L^−1^ KCl, and the N content was determined using an indophenol blue colorimetric method (UV-2102 PCS, Shanghai, China). Total P concentrations of plant and soil were determined colorimetrically with a spectrophotometer (UV-2102 PCS, Shanghai, China). Soil available P was extracted in 0.5 mol L^−1^ NaHCO_3,_ and the P content was determined using the Olsen method. Total K concentrations of plant and soil were determined with a digital flame analyzer (265500, Chicago, Il, USA). Soil available K was extracted in 1 mol L^−1^ NH_4_Ac and then analyzed with a digital flame analyzer (265500, Chicago, IL, USA).

### Leaf and soil nutrient analyses

In this study, nutrient resorption efficiency (NuRE) was calculated on a per plot mass basis using the following equation^51^:

$${\rm{NuRE}}\,( \% )=\frac{{{\rm{Nutrient}}}_{{\rm{gr}}}-{{\rm{Nutrient}}}_{{\rm{sen}}}}{{{\rm{Nutrient}}}_{{\rm{gr}}}}\times 100 \% $$

where Nutrient_gr_ is the nutrient concentration of a mature, green leaf and Nutrient_sen_ is the nutrient concentration of a senesced leaf.

Soil nutrient content (SNC, kg m^−2^) was calculated by multiplying soil nutrient concentration by bulk density and layer thickness^52,53^ as follows:

$${\rm{SNC}}=\frac{{\sum }_{{\rm{i}}=1}^{{\rm{n}}}{{\rm{\rho }}}_{{\rm{i}}}\times {{\rm{C}}}_{{\rm{i}}}\times {{\rm{T}}}_{{\rm{i}}}}{100}$$

where ρ_i_ (g cm^−3^) represents the soil bulk density in soil layer *i* (each 10 cm depth from 0 to 60 cm); C_i_ (g kg^−1^) represents the concentration of the soil nutrient in soil layer *i*; T_i_ (cm) represents the thickness of soil layer *i*; and n represents the total number of soil layers.

### Determination of N, P and K limitation

Stoichiometric ratios of N, P, and K in plant tissue are useful predictors that can often determine which nutrients limit plant growth^54^. In this study, the stoichiometric ratios of N, P, and K in the leaves were calculated on a mass basis. A ternary diagram was constructed in which the type of nutrient limitation is plotted against the specific stoichiometric ratio of N:P:K^37^. In the ternary diagram, each point represents one treatment and dashed lines represent the critical ratios of N:P (14.5), N:K (2.1), and K:P (3.4), dividing the plots into four parts^55^, namely, N limitation (N:P < 14.5 and N:K < 2.1), P limitation or P and N co-limitation (N:P > 14.5 and K:P > 3.4), K limitation or K and N co-limitation (N:K > 2.1 and K:P < 3.4), and nutrient limitation undetermined or no NPK limitation (the central triangle). For example, the opposite axis crossed the point where K = 0% (the horizontal axis), N = 59.2% (the right axis), and 10 P = 40.8%, yielding a critical N:P ratio of 14.5. A point below the line is N-limited; a point above the line is P-limited or P + N-co-limited.

### Statistical analyses

All analyses were performed using SPSS 17.0, and ternary diagrams were drawn using Origin 8.5 software. The effects of P or K fertilization and water supply on soil nutrient contents, nutrient resorption efficiencies, and specific stoichiometric ratios met homogeneity of variance by Levene-test, and were determined using three-way ANOVA. The differences in soil nutrient contents, nutrient resorption efficiencies, and specific stoichiometric ratios among different fertilization treatments under the same water supply or among different water supply levels under the same fertilization treatment were examined using one-way ANOVA followed by Duncan’s multiple range tests and t-tests (*p* < 0.05). The correlations of resorption efficiencies with soil nutrient contents were identified using Spearman’s rank correlation analysis and analyzed with the model *y* = a*x* + b. The correlations of resorption efficiencies with stoichiometric ratios in green leaves were identified using Spearman’s rank correlation analysis and analyzed with the model *y* = a*x*^2^ + b*x* + c.

## Supplementary information

Supplementary Information.

## Data Availability

The datasets generated and/or analyzed during the current study were available from the corresponding author upon reasonable request.
