# Supplementary material for: Leaf Nutrient Resorption in Lucerne Decreases with Relief of Relative Soil Nutrient Limitation under Phosphorus and Potassium Fertilization with Irrigation
Source: Sci Rep. 2020 Jun 29;10:10525. doi: 10.1038/s41598-020-65484-1 (PMC7324584; doi:10.1038/s41598-020-65484-1)
Supplement: Supplementary file 1 — Supplementary Information. [file 41598_2020_65484_MOESM1_ESM.docx]

**Leaf Nutrient Resorption in Lucerne Decreases with Relief of Relative Soil Nutrient Limitation under Phosphorus and Potassium Fertilization with irrigation**

**Mei Yang^1^, Jiaoyun Lu^1^, Minguo Liu^1^, Yixiao Lu^1^, Huimin Yang^1^***

^1^State Key Laboratory of Grassland Agro-ecosystems; Key Laboratory of Grassland Livestock Industry Innovation, Ministry of Agriculture and Rural Affairs; College of Pastoral Agriculture Science and Technology, Lanzhou University, Lanzhou, 730020, P. R. China

***** Corresponding author: *Huimin Yang*

E-mail: [huimyang@lzu.edu.cn](mailto:huimyang@lzu.edu.cn)

**Figure S1.** Effects of P and K fertilization on (a) leaf area, (b) biomass, (c**)** green and (d) senesced leaf K concentrations of lucerne under three water supply level treatments.

LW, low water supply (300 mm year^−1^); NW, normal water supply (450 mm year^−1^); HW, high water supply (600 mm year^−1^); CK, no P or K fertilaiztion. Different capital letters denote significant differences among water supply levels under the same fertilization treatment (*p<*0.05). Different lowercase letters denote significant differences among fertilization treatments under the same water supply level (*p<*0.05). The data are presented as mean±SD.

**Figure S2.** Relationship between senesced leaf (a) N and (b) P concentration and green leaf N:P ratio.

*gr*, green leaf. The vertical dashed gray lines indicate the critical ratio of N:P (14.5)^55^, which divides the figures into left and right sections, representing N-limitation and P or P + N co-limitation, respectively. The horizontal dashed red lines divide the figures into top, middle and bottom sections^46^, respectively representing the nutritional status of senesced leaves: (a) N, <7 mg g^−1^ (complete resorption), >7 but <10 mg g^−1^ (intermediate resorption), and >10 mg g^−1^ (incomplete resorption); (b) P, <0.5 mg g^−1^ (complete resorption), >0.5 but <0.8 mg g^−1^ (intermediate resorption), and >0.8 mg g^−1^ (incomplete resorption).
